# Subgingival microbiota and genetic factors (A-2570G, A896G, and C1196T TLR4 polymorphisms) as periodontal disease determinants

**DOI:** 10.3389/fdmed.2025.1576429

**Published:** 2025-06-09

**Authors:** Raúl Castro-Casarrubias, Natividad Castro-Alarcón, Salvador Reyes-Fernández, Elena Salazar-Hernández, Mirna Vázquez-Villamar, Norma Samanta Romero-Castro

**Affiliations:** ^1^ Facultad de Ciencias Químico Biológicas, Programa de Maestria en Ciencias Biomédicas, Universidad Autónoma de Guerrero, Guerrero, Mexico; ^2^ Laboratorio de Investigación en Microbiología. Facultad de Ciencias Químico Biológicas, Universidad Autónoma de Guerrero, Guerrero, Mexico; ^3^ Department of Implantology and Oral Rehabilitation, Faculty of Dentistry, Autonomous University of Guerrero, Guerrero, México; ^4^ Facultad de Ciencias Agropecuarias y Ambientales, Universidad Autónoma de Guerrero, Guerrero, México

**Keywords:** genetic factors, subgingival microbiota, periodontitis, TLR-4 polymorphism, dysbiosis, periodontal risk factor

## Abstract

**Background:**

Subgingival microbiota play an important role in maintaining oral health. Subgingival dysbiosis leads to the aggregation of highly pathogenic bacteria, and the host’s genetics modulates the innate immune response. The interaction between these two factors plays an important role in the aggravation of periodontitis. Therefore, evaluating the association between the TLR-4 polymorphisms and subgingival microbiota in patients with periodontitis is necessary.

**Methods:**

We included 58 cases with periodontitis and 53 controls without periodontitis in this study. A896G, A-2570G, and C1196T polymorphisms of the *TLR4* gene were determined by the polymerase chain reaction and restriction fragment length polymorphism technique. The DNA–DNA checkerboard hybridization technique was used for the identification and quantification of 18 bacterial species of subgingival plaque.

**Results:**

*Cutibacterium acne*s occurred in greater number and frequency than other bacterial species ($\bar{\chi }$ 1.32 E + 05) in individuals with periodontitis. Patients with *C. acnes* had a higher risk [odds ratio (OR)= 3.82 (95% confidence interval (CI): 1.37–10.3)] of developing periodontitis (*p* < 0.05), as did those with orange and red complex bacteria (*Treponema denticola*, *Tannerella forsythia*, *Porphyromonas gingivalis*, *Prevotella nigrescens*, *Prevotella intermedia, Fusobacterium periodonticum*, *Fusobacterium nucleatum*, *Eubacterium nodatum,* and *Campylobacter rectus*). The A/G genotype of SNP -2570 of the *TLR4* gene was identified as a risk factor for the development of periodontitis [OR = 2.28 (95% CI: 1.04–5.00)]. Furthermore, there was an antagonistic biological effect of the presence of bacteria such as *Capnocytophaga gingivalis* [OR = 0.44 (95% CI: 0.20–1.96)] and *C. rectus* [OR = 0.39 (95% CI: 0.18-0.87)] (*p* < 0.05). The A/G genotype of SNP-2570 was correlated with greater clinical attachment loss and periodontal pocket depth.

**Conclusions:**

The agonistic or antagonistic biological effect of each bacterial species depends on the genotype present in each individual and the destruction processes of dental support tissues.

## Introduction

Periodontitis is a chronic infection that is considered a non-communicable disease and is induced by the constant challenge of a polymicrobial dysbiotic biofilm in the presence of a dysregulated immune response in a genetically susceptible host (1). This is characterized by the destruction of the periodontal ligament, alveolar bone, and epithelial tissue that form part of the dental support (2). Forming the biofilm favors the multiplication of pathobionts and opportunistic bacteria (3). The subgingival biofilm located in the subgingival sulcus is commonly composed of Gram-negative anaerobic bacteria such as *Porphyromonas gingivalis, Tannerella forsythia,* or *Treponema denticola*, whose membrane is structurally made up of lipopolysaccharides (LPSs) (4). LPS binding to Toll-like receptor 4 (TLR4) initiates the innate immune response and induces an inflammatory response in subgingival epithelial tissues through signaling cascades (5). When a TLR4/MD2 heterotetrameric is formed (6, 7), the pathway dependent on the myeloid differentiation primary response protein (MyD88) and the endosomal pathway can converge and potentiate the transcription of proinflammatory cytokines from the activation of TRAF6 by the dimer between TRAM and TRIF (8). The constant stimulation of inflammation by proinflammatory cytokines (IL-1-β, IL-6, IL-11, IL-17, and TNF-α) can induce osteoclastogenesis by increasing the expression of RANKL, decreasing the production of osteoprotegerin (9), and increasing the destruction of dental support tissues, which is typical of periodontitis (1, 2).

The single-nucleotide polymorphisms (SNPs) in the *TLR4* gene are associated with an attenuated response to LPS exposure in humans. Two of the most common co-segregating SNPs causing receptor extracellular domain shortening are the A896G (Asp-299Gly) and C1196T (Thr399Ile) polymorphisms (10, 11). SNP A-2570G causes the loss of binding sites for the transcription factors Tst-1 and CdxA and generates a binding site for v-Myb, which represses promoter activity in the presence of infection or exposure to LPS (12). The protective or risk effect of these polymorphisms with subgingival dysbiosis and periodontitis has not been analyzed.

In Mexico, in 2023, 167,611 patients were treated in health centers and hospitals, of which 2.1% were between 20 and 34 years old and 5.3% were diagnosed with periodontitis (13). In the State of Guerrero, the prevalence of periodontal diseases was 82% in 2016 (14). In order to understand how periodontal diseases begin and develop, it is essential to know and characterize the bacterial profiles present in our population and the frequency of the A-2570G, A896G, and C1196T polymorphisms of the *TLR4* gene and their association with periodontitis.

## Materials and methods

### Selection of participants, study design, and bioethical considerations

All the patients who received preventive or curative treatment in the period from August 2019 to June 2020 at the Periodontics Clinic belonging to the Faculty of Dentistry of the Autonomous University of Guerrero were invited to participate in the study, and only those who agreed to the study and signed their consent form were included. To establish the periodontal diagnosis, patients over 18 years old with at least 20 permanent teeth in their oral cavity were included. Patients receiving antibiotic treatment or anti-inflammatory or immunosuppressive drugs for at least 3 months before the study were excluded. In addition, patients with inflammatory, metabolic, or chronic degenerative diseases and pregnant or lactating women were also excluded. All individuals voluntarily agreed to participate in the study by signing an informed consent letter. The study adhered to the Declaration of Helsinki for the World Medical Society (15), and the Ethics and Research Committee of the Autonomous University of Guerrero approved the project.

### Clinical and radiographic evaluation

Bone loss was assessed by dentoalveolar projections (periapical radiography). Clinical parameters such as probing depth (PD), levels of clinical attachment loss (CAL), and bleeding on probing (BOP) were taken with the periodontal probe (Florida Probe Corporation, USA). All the patients were probed by a single operator who had been trained by an experienced periodontist. Furthermore, the electronic probing method chosen reduced measurement errors, even by the same operator. The clinical parameters of furcation and mobility compromises were also considered, as was the plaque index, which was determined by the O'Leary method (16).

### Patient classification

The groups were formed as follows.

#### Control group

Patients with good periodontal health or some degree of gingivitis were enrolled in the control group, as it was important to understand the genetic predisposition and microbial interactions that disrupt this balance and trigger destructive disease.

#### Case group

Patients with destructive periodontal disease at any stage (periodontitis) were enrolled in the case group.

Those who were considered healthy were without bleeding on probing or clinical changes in the gingiva, including patients with a healthy periodontium and clinically healthy gingiva in a reduced periodontium in a patient without periodontitis.

Those with bleeding on probing and clinical changes in the gums in the following conditions were considered to have gingivitis: intact periodontium, reduced periodontium in a patient without periodontitis, and reduced periodontium in patients with successfully treated and stable periodontitis.

To define the patients with periodontitis, the reclassification of periodontitis accepted in 2017 by The World Workshop on the Classification of Periodontal and Peri-implant Diseases was taken into account due to the small sample size, and in order not to lose statistical power in the analysis, the patients with periodontitis were not stratified. Those with destructive disease were grouped as follows: interdental CAL at site of greatest loss: 1–2 mm=Stage I; 3–4 mm Stage II; ≥5 mm and tooth loss due to periodontitis of <4=Stage III; ≥5 mm and tooth loss due to periodontitis of ≥5=Stage IV (17).

### Obtaining subgingival plaque

Patients were asked not to brush their teeth the night before the study and to come early in the morning after fasting. Relative isolation was performed with cotton rolls and air-dried with a triple syringe. Supragingival plaque was removed, avoiding bleeding, and finally, a pooled subgingival plaque sample was collected with a sterile curette (Gracey mini-five no. 11/12). The sample was taken from the first four molars or, alternatively, from the second molars, as these molars are considered to have a higher amount of representative pathogenic microbiota due to their inaccessibility when brushing. Sample plaques were deposited in a microtube with 1.5 ml of vehicle, 150 µl of TE buffer (10 mM Tris-HCl and 0.1 mM EDTA), and 100 µl of NaOH (0.5 M) for subsequent storage at −20°C (18).

### Blood sampling

Blood sampling was carried out in the Faculty of Dentistry of the Autonomous University of Guerrero by a laboratory technician who had been trained by a leading laboratory chemist. Patients were asked to come in after fasting in the early hours of the morning and 5 ml of blood was collected by venipuncture and placed in EDTA tubes (BD Vacutainer®) to obtain DNA.

### Identification and quantification of the subgingival microbiota

Identification and quantification of the subgingival microbiota were performed using the checkerboard DNA–DNA hybridization technique at the Laboratory on Molecular Genetics in the Faculty of Stomatology of the National Autonomous University of Mexico, Mexico (LGM-UNAM). DNA–DNA hybridization was used to analyze the samples individually using the DNA–DNA checkerboard hybridization technique. Initially, the samples were thawed at room temperature, boiled for 10 min, and then neutralized with 800 ml of 5 M ammonium acetate. The DNA released from each sample was placed in individual lanes and concentrated on a positively charged 15 × 15 cm nylon membrane, which was fixed by cross-linking under ultraviolet light. Two lanes of each membrane contained standards consisting of a mixture of 10^5^ and 10^6^ cells of each bacterial species tested. Subsequently, the membranes were prehybridized at 42°C for 2 h in a solution composed of 50% formamide, 5× standard saline citrate (SSC) (1× SSC = 150 mM NaCl and 15 mM Na citrate; pH 7.0), 1% casein, 5× Denhardt's solution, 25 mM sodium phosphate (pH 6.5), and 0.5 mg/ml of yeast RNA. Then, each membrane was placed in a device in which the lanes with the samples were rotated 90° in relation to the channels of the apparatus. The probes were diluted to approximately 20 ng/ml in a hybridization solution containing 45% formamide, 5× SSC, 1× Denhardt's solution, 20 mM sodium phosphate (pH 6.5), 0.2 mg/ml yeast RNA, 10% dextran sulfate, and 1% casein.

These probes were placed in individual device channels and hybridized overnight at 42°C. The probes were hybridized in four sets of 10 consecutive channels, leaving one empty channel (with hybridization solution only) between each set to allow for the correction of noise and background signals. The membranes were washed twice at high stringency for 20 min at 68°C in phosphate buffer (0.1× SSC and 0.1% SDS). Regarding taxon detection and enumeration, membranes were incubated for 1 h in a blocking buffer containing 1% casein in maleate buffer (100 mM maleic acid and 150 mM NaCl, pH 7.5). Hybrids were detected by exposing the membranes to a 1:50,000 dilution of anti-digoxigenin antibody conjugated with alkaline phosphatase for 30 min. Signals were detected by chemiluminescence. Briefly, the membranes were incubated with a chemiluminescent agent for 5 min at room temperature and then exposed to film in autoradiographic cassettes for 10 min. The films were developed and photographed using a digital photodocumentation system. The signals were detected and analyzed with specialized software, adjusting the results by subtracting the average plus two standard deviations of the noise and background detected in the three empty lanes and converting them into absolute counts by comparison with the standards for each membrane. In cases where no signal was detected, a value of zero was recorded. The detection limits of the technique to detect the standards of 10^5^ and 10^6^ were calculated by linear regression to a minimum detection limit of 10^4^ and a maximum of 10^7^ cells (19).

Probes were used to identify the following 18 bacterial species: *Actinomyces georgiae, Actinomyces naeslundii, Aggregatibacter actinomycetemcomitans b, Campylobacter rectus, Capnocytophaga gingivalis, Capnocytophaga sputigena, Eubacterium nodatum, Fusobacterium nodatum, Fusobacterium nucleatum, P. gingivalis, Prevotella intermedia, Prevotella nigrescens, Cutibacterium acnes, Streptococcus anginosus, Streptococcus gordonii, Tannerella forsythia, Treponema denticola,* and *Veillonella parvula*.

### DNA extraction

Genomic DNA (gDNA) extraction was performed using peripheral blood leukocytes from each blood sample, using the modified Miller technique as previously reported (20), as follows. Buffy coats of nucleated cells obtained from anticoagulated blood (ACD or EDTA) were resuspended in 15 ml polypropylene centrifugation tubes with 3 ml of nuclei lysis buffer (10 mM Tris-HCl, 400 mM NaCl, and 2 mM Na2EDTA, pH 8.2). The cell lysates were digested overnight at 37°C with 0.2 ml of 10% SDS and 0.5 ml of a protease K solution (1 mg protease K in 1% SDS and 2 mM Na2EDTA). After digestion was complete, 1 ml of saturated NaCl (approximately 6 M) was added to each tube and shaken vigorously for 15 s, followed by centrifugation at 2,500 rpm for 15 min. The precipitated protein pellet was left at the bottom of the tube and the supernatant containing the DNA was transferred to another 15 ml polypropylene tube. Exactly 2 volumes of room temperature absolute ethanol were added and the tubes were inverted several times until the DNA precipitated. The precipitated DNA strands were removed with a plastic spatula or pipette and transferred to a 1.5 ml microcentrifuge tube containing 100–200 μl TE buffer (10 mM Tris-HCl, 0.2 mM Na2EDTA, pH 7.5). The DNA was allowed to dissolve for 2 h at 37°C before quantitating.

The DNA obtained from this simple technique yielded quantities comparable to those obtained from phenol-chloroform extractions. The 260/280 ratios were consistently 1.8–2.0. Finally, DNA concentration was performed using a Nanodrop 2000C (ThermoFisher Scientific, Wilmington, USA). The minimum purity of DNA to be used was 1.8 a 1.9 ratios.

### PCR *TLR4* gene polymorphisms

The TLR4 polymorphisms were genotyped using the polymerase chain reaction and restriction fragment length polymorphism (PCR–RFLP) technique (11, 12, 21). The regions containing the 896 A > G (Asp-299Gly, rs4986790), 1196 C > T (Thr399Ile, rs4986791), and −2570 A > G (rs2737190) polymorphisms were amplified by PCR, and a pair of specific primers was used for each polymorphism. The reaction mixture to determine each polymorphism was performed in a final volume of 25 μl using 1× of GO Taq® Green Master Mix (Promega), 0.5 μM of each oligonucleotide, 100 ng of gDNA, and adjusting the final volume with H_2_O. The following cycling conditions were used: initial denaturation at 94°C for 10 min, 35 cycles of 94°C for 30 s for denaturation, 60°C for 30 s for annealing, extension at 72°C for 30 s, and 72°C for 7 min for an ending extension. The PCR-amplified fragments were observed on a 6% polyacrylamide gel electrophoresis at 90 V for 1 h and stained with silver nitrate. The specific primers and the amplification size are described in Table 1.

**Table 1:** Oligonucleotides used for DNA amplification.

Polymorphism	Primer	Amplifier size (bp)
A-2570G	F: 5´-TGGTACCTGGACCTGTGATGAT-3´ R: 5´-GTTCCCTGGAAAGTTAATGGTGT-3´	334
A896G	F: 5´-GATTAGCATACTTAGACTACTACCTCCATG-3´ R: 5’-GATCAACTTCTGAAAAAGCATTCCCAC-3´	249
C1196T	F: 5´-GGTTGCTGTTCTCAAAGTGATTTTGGGAGAA-3´ R: 5´-CCTGAAGACTGGAGAGTGAGTTAAATGCT-3´	405

### Enzymatic restriction

The PCR products were digested with restriction enzymes to determine the polymorphisms. For 896 A > G, we used NCoI; for A-2570G, we used TspRI; and for C1196T, we used HinfI (New England Biolabs, Ipswich, Massachusetts, USA). One microliter of each PCR product was digested with the respective restriction enzymes (0.2 µl endonuclease and 1 µl buffer) in a final volume of 10 µl and incubated at 37°C (NCoI, HinfI) or 65°C (TspRI) for 30 min.

The restriction patterns generated for the 896 A > G polymorphism were 249 bp for AA; 249, 223, and 26 bp for AG; and 223 and 26 bp for the GG genotype. For the 1196 C > T polymorphism, the digested fragments were 405 bp for CC; 405, 376, and 29 bp for CT; and 376 and 29 bp for the TT genotype. For the −2570 A > G polymorphism, the digested fragments were 334 bp for AA and 334, 293, and 41 bp for AG. Electrophoresis was performed in 7% (29:1) polyacrylamide gels to visualize them, and the samples were run at 120 V for 1 h (22).

### Statistical analysis

Data were analyzed using the statistical program Stata 15® for Windows ® (StataCorp, College Station, TX, USA). The qualitative variables were reported in relative frequencies, and the study groups were compared using the *χ*^2^ test or Fisher’s test. By this same method, the allelic frequencies and the distribution of the genotypes were analyzed. The normal distribution variables were reported using the mean ± standard deviation, and the analysis of these data was performed using Student’s *t*-test. In the case of non-symmetrical quantitative variables, medians with percentiles (25–75) were reported, and the comparison between the two groups was made using the Mann–Whitney test.

The odds ratio (OR) was calculated with a 95% confidence interval (CI) using logistic regression models to determine the associations of the polymorphisms in both study groups. To calculate the probability of finding at least one polymorphism, the online program http://www.OpenEpi.com V3.01 (23) was used, in which population data for the A-2570G, A896G, and C1196T polymorphisms of the *TLR4* gene in Mexico were found (21). To determine the allelic, genotypic, and haplotypic frequencies, the SHEsis® online program was used (24, 25). We carefully reviewed the behavior of the three SNPs of the *TLR4* gene in the databases of the ALFA project and the 1,000 Genomes Project. Results were considered significant with a *p* < 0.05 in a two-tailed test.

## Results

### Clinical and sociodemographic characteristics

Of the 111 patients in this study, 58 were classified as cases (patients with periodontitis) and 53 as controls (individuals without periodontal disease or with gingivitis). In this study, we observed that older people tended to have more frequent periodontitis compared to younger people (*p* < 0.001), and a low level of education was associated with having periodontitis (*p* < 0.003). The clinical characteristics evaluated (CAL and PD, BOP, and plaque index) were higher in the patients with periodontitis. Smoking status showed no significant association with periodontitis disease (Table 2).

**Table 2:** Analysis of the study groups according to clinical and sociodemographic characteristics.

Characteristic	Total *n* (100%)	Without periodontitis^c^, *n* (48%)	With periodontitis^d^, *n* (52%)	*p*
Age (groups)				
Young, *n* (%)	63 (56.8)	45 (71.4)	18 (28.6)	**<0**.**001**
Adult, *n* (%)	41 (36.9)	8 (19.5)	33 (80.5)	
Old age, *n* (%)	7 (6.3)	0 (0)	7 (100)	
Sex, *n* (%)				0.16^b^
Female	43 (38.74)	36 (68)	32 (55)	
Male	68 (61.26)	17 (32)	26 (45)	
Education, *n* (%)				**0**.**003**^b^
College	56 (50.45)	35 (66)	21 (36)	
High school	21 (18.92)	10 (19)	11 (19)	
Junior high school	12 (10.81)	5 (9)	7 (12)	
Primary	18 (16.22)	3 (6)	15 (26)	
Without education	4 (3.60)	0 (0)	4 (7)	
Plaque index (%)	30 (17.4–51)	21 (11–51.3)	33 (26–50.8)	**0**.**01**^a^
BOP (index)	4 (0–15)	0 (0–4)	14 (0–15)	**<0**.**001**^a^
PD (mm)	4 (0–4)	0 (0–0)	4 (4–4.3)	**<0**.**001**^a^
CAL (mm)	4 (0–4.3)	0 (0–3)	4.3 (4–4.6)	**<0**.**001**^a^
Smoking (cigarettes/day)				0.76^b^
0	98 (88.29)	46 (86.79)	52 (89.66)	
1–2	10 (9.01)	5 (9.43)	5 (8.62)	
3–4	2 (1.80)	1 (1.89)	1 (1.72)	
≥5	1 (0.90)	1 (1.89)	0 (0)	

For the analysis by age groups and tobacco consumption, Fisher's exact test was used. *P*-values <0.05 were considered statistically significant. BOP, bleeding on probing; PD, probing depth; CAL, clinical attachment level. .

Bold value indicates statistically significant results.

^a^ Plaque index, BOP, PD, and CAL are presented in p50 (p25–p75), and the *p*-value was calculated by the Mann–Whitney test.

^b^ Categorical variables are shown in percentages (%), and the *p*-value was calculated by the X^2^ test.

^c^ Without periodontitis: healthy and gingivitis.

^d^ With periodontitis (Stages I, II, or III, and Grades A, B, or C).

### Subgingival microbiota

In the analysis of the 18 bacterial species of subgingival plaque, it was found that there was a higher frequency of *P. intermedia* (74.77%), *T. forsythia* (71.21%), *A. actinomycetemcomitans b* (21.25%), and *P. nigrescens* (75.68%) in individuals with periodontitis. *A. naeslundii* (48.84%) and *A. georgiae* (47.54%) showed a low frequency among the study groups. The frequency of *C. acnes* was 76.92%, and was a bacterium found to have a probable influence on the worsening of periodontitis, with *p-*values < 0.05 (not shown graphically).

**Figure 1:**
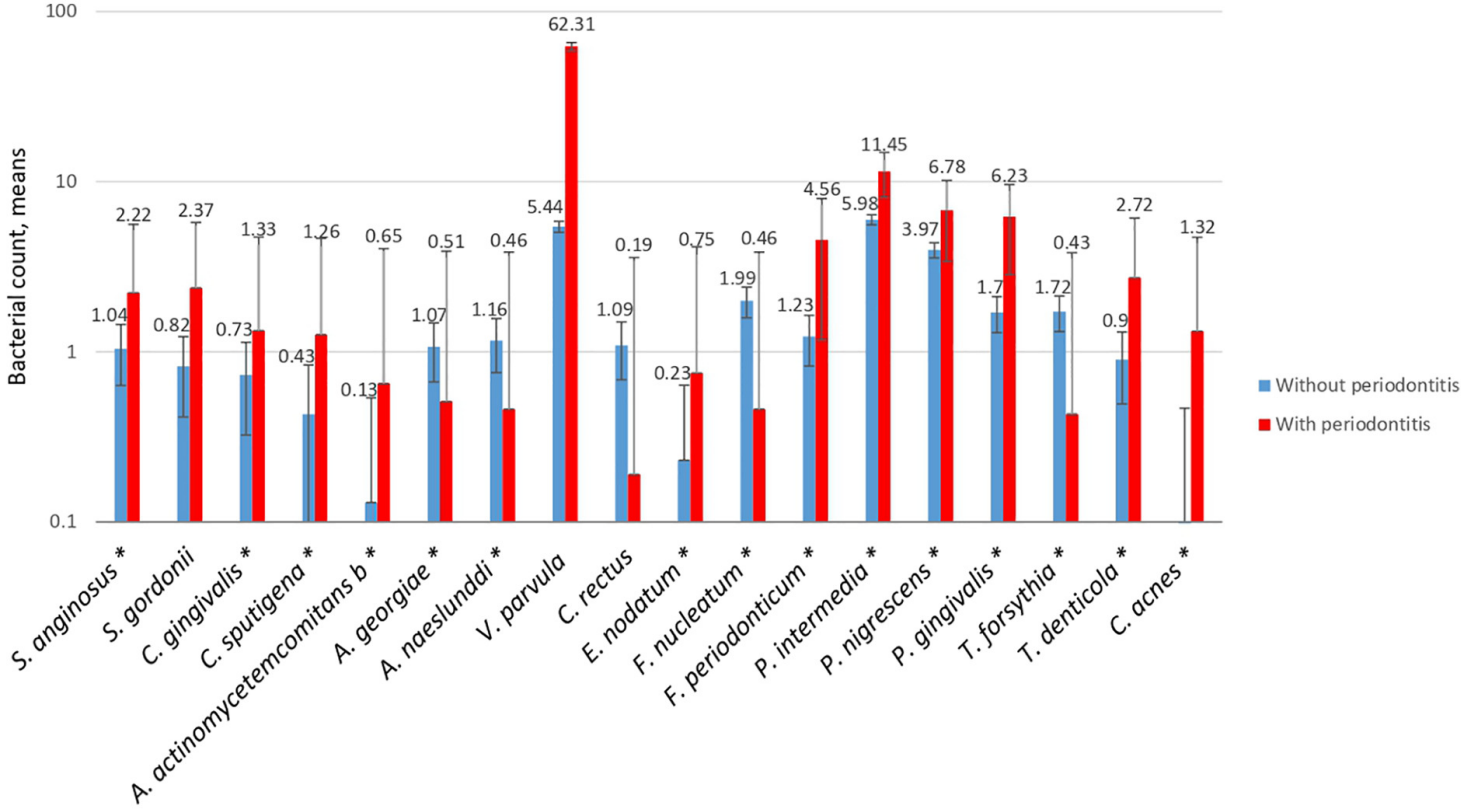
Mean count of the bacterial species in subgingival plaque in both study groups with an E + 05. The red line refers to the cases and the blue line to the controls. A *p*-value of <0.05 was considered statistically significant (*), as calculated using logistic regression.

Mean bacterial counts were determined in the groups with and without periodontitis, and then a logistic regression analysis was performed to determine whether the quantitative frequency was statistically significant. The logarithmic scale of the bacterial count was 10E + 05 (Figure 1). With bacterial species such as *V. parvula* and *C. rectus*, no association was observed in the qualitative frequency analysis or the determination of mean bacterial counts.

### *TLR4* gene polymorphisms

Subsequently, the genotyping of the A-2570G, A896G, and C1196T polymorphisms of the *TLR4* gene was performed, and the genotypic and allelic frequencies were determined (Table 3). For the A-2570G polymorphism, it was found in genotypes A/A, A/G, and G/G; for the A896G polymorphism, the A/A and A/G genotypes were found, but not the polymorphic homozygote (G/G). For the C1196T polymorphism, only the C/C and C/T genotypes were found. The three TLR4 gene polymorphisms were in Hardy–Weinberg equilibrium (*p* > 0.05). A logistic regression model was used to determine whether the polymorphisms provided a biological effect in individuals with and without periodontitis, and, to calculate the risk, ORs were calculated, showing that the A/G genotype of SNP -2570 was a risk factor (OR = 2.1, CI 95% 1.00–3.65, *p* < 0.05), with a sex-adjusted OR of 2.28 (95% CI 1.04–5.00, *p* < 0.05) (Table 4).

**Table 3:** Genotypic and allelic frequencies of polymorphisms A-2570G (rs2737190), A896G (rs4986790), and C1196T (rs4986791) of the *TLR4* gene in individuals with and without periodontitis.

Polymorphism	Without periodontitis, 53 (47.75)	With periodontitis, 58 (52.25)	HWE	OR (CI 95%)	*p*
−2570 A > G					
Genotype			X^2^ (2.2) *p* (0.13)	2.1 (1.00–4.65) 1.38 (0.081–23.4)	0.049
A/Aª	29 (26.13)	21 (18.92)			
A/G	23 (20.72)	36 (32.43)			
G/G	1 (0.90)	1 (0.90)			
Allele					
A	81 (76.4)	78 (67.24)			
G	25 (23.6)	38 (32.76)			
896 A > G					
Genotype			X^2^ (0.04) *p* (0.83)	0.29 (0.02–2.90)	0.29
A/Aª	50 (45.05)	57 (51.35)			
A/G	3 (2.70)	1 (0.90)			
G	0	0			
Allele					
A	103 (97.17)	115 (99.14)			
G	3 (2.83)	1 (0.86)			
1196 C > T					
Genotype					
C/C^a^	50 (45.05)	57 (51.35)	X^2^ (0.04) *p* (0.83)	0.29 (0.02–2.90)	0.29
C/T	3 (2.70)	1 (0.90)			
T	0	0			
Allele					
C	103 (97.17)	115 (99.14)			
T	3 (2.83)	1 (0.86)			

The data are expressed in *n* (%); *p* < 0.05 was considered statistically significant. A logistic regression model was used to determine the ORs. OR, odds ratio; CI, confidence interval; HWE, Hardy–Weinberg Equilibrium. Genetic balance was considered with a value of *p* > 0.05.

^a^ Reference genotype.

**Table 4:** Associations of polymorphisms A-2570G, A896G, and C1196T of the *TLR4* gene in individuals with and without periodontitis.

Haplogenotype	Without periodontitis, *n* (%), 53 (47.75)	With periodontitis, *n* (%), 58 (52.25)	OR	CI 95%	*p*
−2570 A > G (rs2737190)					
AA^a^	29 (26.13)	21 (18.92)			
AG	23 (20.72)	36 (32.43)	**2**.**28**	**1.04** **–** **5.00**	**0**.**03**
GG	1 (0.90)	1 (0.90)	1.32	0.075–23.16	0.48
896 A > G (rs4986790)					
AA^a^	50 (45.05)	57 (51.35)			
AG	3 (2.70)	1 (0.90)	0.35	0.03–3.59	0.38
GG	0 (0)	0 (0)			
1196 C > T (rs4986791)					
CC^a^	50 (45.05)	57 (51.35)			
CT	3 (2.70)	1 (0.90)	0.22	0.02–2.33	0.21
TT	0 (0)	0 (0)			

Logistic regression model. OR, odds ratio; CI, confidence interval. *P* < 0.05 was considered statistically significant.

Bold value indicates statistically significant results.

^a^ Reference genotype.

To analyze the correlation between the A-2570G, A896G, and C1196T polymorphisms of the *TLR4* gene and the clinical parameters of periodontal disease, a linear regression model was used, with the crude data adjusted for age, sex, and education (data not shown). The A/G genotype of SNP-2570 was correlated with the levels of periodontal pocket depth (*β* 0.84, *p* < 0.05) and clinical attachment loss (*β* 1.06, *p* < 0.05); however, the wild-type A/A genotype of the SNP-2570 was negatively correlated with periodontal pocket depth (*β* −0.82, *p* < 0.05) and clinical attachment loss (*β* −1.02, *p* < 0.05). Furthermore, the wild-type A/A genotype of SNP 896 was negatively correlated with the plaque index (β −21.6, *p* < 0.05) and, after adjusting the data for age, sex, and schooling, we observed that the A/G genotype of SNP A896G was positively correlated with the plaque index (β 21.6, *p* < 0.05).

### Association between polymorphisms and subgingival microbiota

To analyze whether the bacterial species found in the subgingival biofilm can exert a biological effect on individuals with the genotypes of the SNPs A-2570G, A896G, and C1196T of the *TLR4* gene, a logistic regression model was used to determine the ORs (Table 5). The evidence suggests that the host's genotype was a determinant of a protective biological effect or risk against a bacterium in the subgingival plaque, regardless of bacterial serotype. Our study suggests that a single bacterium, *S. anginosus,* had an antagonistic role, that is, it had a protective effect for the A/A genotype and a risk effect for the A/G genotype of SNP A-2570G. A similar case occurred for *S. gordonii*, which is considered an initial colonizing bacterium in the subgingival plaque. In the case of the A/A genotype, not all the initial colonizing bacteria played a protective role; the presence of *C. rectus* had a risk effect, contrary to the protective effect of hosts that carry the A/G genotype of SNP A-2570G against bacteria such as *C. gingivalis* and *C. rectus*. Although the aggregation of pathogenic bacteria aggravates periodontitis, the sum of factors, such as genetic predisposition in individuals with the A/G genotype of SNP A-2570G, can trigger a more destructive scenario for supporting tissues in the presence of bacteria such as *C. acnes*. The biological role of *C. acnes* is uncertain; however, it has a protective effect in individuals with the A/A genotype of SNP A-2570G of the *TLR4* gene.

**Table 5:** Correlations between polymorphisms A-2570G, A896G, and C1196T of the *TLR4* gene and the presence of the 18 bacterial species.

Complex	Bacterial species	TLR4-2570 A > G genotype					
		AA		AG		GG	
		OR (IC 95%)	*p*	OR (IC 95%)	*p*	OR (IC 95%)	*p*
Yellow	*S. anginosus*	**0.27** (**0.12–0.62)**	**0**.002*	**3.73** (**1.66–8.38)**	**<0**.001*	1.27 (0.07–21.51)	0.86
Yellow	*S. gordonii*	**0.41** (**0.18–0.91)**	**0**.02*	**2.58** (**1.17–5.70)**	**0**.01*	–	–
Blue	*A. georgiae*	0.80 (0.37–1.70)	0.57	1.2 (0.54–2.49)	0.68	–	–
Blue	*A. naeslundii*	0.94 (0.43–2.03)	0.88	0.97 (0.45–2.11)	0.93	–	–
Purple	*V. parvula*	1.11 (0.50–2.47)	0.78	0.99 (0.44–2.22)	0.98	–	–
Orange	*F. nucleatum*	0.71 (0.33–1.51)	0.37	1.43 (0.66–3.08)	0.35	0.85 (0.05–14.39)	0.91
Orange	*F. periodonticum*	0.87 (0.39–1.94)	0.74	1.17 (0.52–2.62)	0.69	0.51 (0.03–8.75)	0.64
Orange	*P. intermedia*	0.52 (0.21–1.23)	0.14	2.05 (0.84–4.97)	0.11	0.47 (0.02–8.01)	0.60
Orange	*E. nodatum*	0.51 (0.23–1.13)	0.09	2.05 (0.93–4.49)	0.07	–	–
Orange	*P. nigrescens*	0.69 (0.29–1.66)	0.41	1.52 (0.62–3.68)	0.35	0.38 (0.02–6.65)	0.51
Orange	*C. gingivalis*	2.09 (0.97–4.50)	0.06	**0.44** (**0.20–0.96)**	**0**.03*	–	–
Green	*C. rectus*	**2.50** (**1.14–5.45)**	**0**.02*	**0.39** (**0.18–0.87)**	**0**.02*	0.47 (0.02–8.01)	0.60
Green	*C. sputigena*	0.63 (0.28–1.38)	0.25	1.56 (0.71–3.43)	0.26	2.1 (0.12–36.18)	0.60
Red	*P. gingivalis*	0.87 (0.39–1.94)	0.74	1.17 (0.52–2.62)	0.69	0.51 (0.03–8.75)	0.64
Red	*T. forsythia*	0.89 (0.41–1.91)	0.77	1.13 (0.52–2.44)	0.74	−0.22 (0.42–12.24)	0.82
Red	*T. denticola*	0.85 (0.40–1.81)	0.68	1.24 (0.58–2.66)	0.56	–	–
Not grouped	*A. actinomicetemcomitans b*	1.26 (0.43–3.64)	0.66	0.70 (0.23–2.10)	0.53	5.25 (0.29–92.87)	0.25
Other	*C. acnes*	**0.27** (**0.10–0.76)**	**0**.01*	**3.76** (**1.37–10.3)**	**0**.01*	–	–

Logistic regression model, OR analysis with 95% CI; OR, odds ratio; CI, confidence interval.

Bold value indicates statistically significant results.

* *p-*values of  <0.05 were considered statistically significant.

### Haplogenotypic frequency and linkage disequilibrium

An analysis of the genotypic and haplotype frequencies of the *TLR4* gene was performed in both study groups. Table 6 shows the genotypes for the *TLR4* gene with frequencies > 1%. Other haplogenotypes were found, such as AAAACT, AAGGCC, AGAACC, AGAACT, AGAGCC, and AGGGCC, the frequencies of which were <1%, and we decided to group them into the Other group. The AAC haplotype had a frequency of 72.7% in the controls and 37.2% in the cases. The second most frequent haplotype was AGT, with 22.6% in the controls and 31% in the cases. Haplotypes with a frequency greater than 1% (AAT and GAC) were considered. The AGT, GAT, and GGC haplotypes had a frequency of less than 1%. The linkage disequilibrium (LD) of the three polymorphisms of the *TLR4* gene in the control group was calculated. When comparing the A896G and A-2570G polymorphisms, we found an LD of 0.826 (82), and between A-2570G and C1196T, we found an LD of 0.826 (82); it is important to mention that there was no linkage disequilibrium between the A-2570G polymorphism and C1196T (data not plotted).

**Table 6:** Frequency of haplogenotypes of polymorphisms A896G, A-2570G, and C1196T of the *TLR4* gene in both study groups.

Haplogenotype	With periodontitis	Without periodontitis	*p*	OR (CI-95%)	*p*
AA AG CC	58 (60.3%)	42 (39.6%)	9.51*	**2.3** (**1.3–3.9)**	**0**.**002**
AA AG CT	2 (1.7%)	2 (1.9%)	**0**.008*	0.9 (0.1–6.5)	0.92*
AA AA CC	42 (36.2%)	52 (49.1%)	3.7*	0.5 (0.3–1.0)	0.05*
Others†	4 (3.4%)	10 (9.5%)	0.13	–	–

Logistic regression to determine the ORs.

Bold value indicates statistically significant results.

* A *p-*value calculated using Fisher's test and the∼*X*^2^ test <0.05 was considered statistically significant. OR, odds ratio; CI, confidence interval.

† Genotypes with low frequency. The results show the number of haplotypes and the percentage of the population it represents.

## Discussion

TLR4 activation by Gram-positive bacteria is still a matter of debate; however, we know they possess a thick layer of peptidoglycan and lipoteichoic acid (LTA) that can stimulate TLR4, for example. Species such as *Staphylococcus aureus* and *Streptococcus pneumoniae* have been observed to interact with the TLR4/MD-2/CD14 complex, activating pathways similar to those activated by LPS from Gram-negative bacteria (26). Bacterial replication leaves fragments in the host extracellular matrix that can be recognized by TLR4, inducing the production of cytokines such as TNF-α, IL-6, and IL-1β (27). The immunomodulation generated from the interaction of the TLR4 of dendritic cells and macrophages with the heat shock proteins (HSP60 and HSP70) of *S. aureus* and *S. pneumoniae* suggests a relevant role in the activation of NF-*κ*B and MAPK and cellular stress, promoting an inflammatory response (28). Therefore, it is suggested that bacteria such as *C. acnes* (29), *A. georgiae*, *A. naeslundii* (30), *S. anginosus*, *S. gordonii* (31), and *E. nodatum* can activate TLR4 through the release of HSPs during oral infections and under specific conditions such as replication (32).

In this study, we found that heterozygous carriers of SNP-2070 A > G were associated with periodontitis and the presence of *C. acnes*, *S. anginosus,* and *S. gordonii*. Our results showed that periodontitis was more frequent in older people than in younger people. The oral modifications that lead to age-related periodontitis can have two origins: aging or as a consequence of the accumulation of physiological factors that induce functional and structural biochemical changes (33), in addition to external agents that make one more susceptible to periodontitis.

In this study, it was found that a low level of education was associated with having periodontitis (*p* < 0.05), and this could have been due to different reasons, including a lack of access to information and a lack of interest in knowing preventive measures or treatments for periodontal disease (34).

Although no direct association was found between smoking and periodontitis in our study, smoking has been identified as an important risk factor in the development and progression of periodontal disease. Alterations in the subgingival microbiota facilitate the acquisition and early colonization of periodontal pathogens, leading to dysbiosis in certain oral sites such as subgingivally (35).

As expected, the clinical parameters that are characteristic of periodontitis (PD and CAL) indicated a higher rate of destruction and the accumulation of dental bacterial plaque. The induction of mechanical stimuli through the maturation of the biofilm on the crevicular epithelial tissue contributes to bleeding in individuals with periodontal disease. The results found in the present study on the subgingival microbiota of patients from the state of Guerrero with and without periodontitis are similar to those previously reported in the microbial complexes in subgingival plaque (36), except for the bacterium *C. acnes*, which is usually found within the sebaceous follicles of humans. This bacterium metabolizes fatty acids (37), which explains its presence in the oral cavity, since as the biofilm matures, there is a greater deposit of degraded food debris, such as short-chain fatty acids, that serve as nutrients for *C. acnes*, favoring bacterial replication, and with this, the exacerbation of the inflammatory response against *C. acnes*.

Theoretically, the progression of periodontitis is attributed non-specifically to the result of hypersensitivity, hyper-response, and/or lack of a sufficient resolution of inflammation (38). For this reason, analyzing genetic factors such as polymorphisms that contribute to or modify the inflammatory response allowed us to discuss the biological effects on the receptors that recognize Gram-negative bacterial endotoxins. Obesity, considered a low-grade systemic chronic disease, has a significant impact on the modulation of the immune response, which in turn induces changes in the composition of the microbiota in various regions of the body, including the mouth. Furthermore, during aging, the immune system enters a state known as inflammaging, characterized by low-grade systemic inflammation, which predisposes individuals to the development of inflammatory diseases such as periodontitis (39). The determination of the A-2570G polymorphism of the *TLR4* gene in obese patients indicates that the heterozygous and homozygous genotypes do not present any association (40). Due to this, in our study, obesity was excluded.

However, GG/AA + AG was associated with moderate/severe chronic periodontitis in a Chinese population infected with *P. gingivalis* (41) in the urinary tract, with the A/G and G/G genotypes as risk factors. These results are similar to those found in our study since the presence of the A/G genotype increased the probability of developing periodontitis and increased clinical attachment loss and pocket depth. This biological effect may be due to the fact that the presence of the A-2570G polymorphism potentially affects a binding site for CdxA, a homeodomain protein, and Tst-1, which is a POU (Pit-Oct-Unc) transcription factor that significantly decreases the expression of the *TLR4* gene (12), leading to chronic exposure to proinflammatory cytokines.

The repression effect of the *TLR4* gene promoter provides a protective factor against bacteria such as *C. gingivalis* and *C. rectus*, which are Gram-negative bacteria that could exacerbate local inflammation due to their LPS in the crevicular epithelial tissue with the wild-type A/A genotype. Although LPS from Gram-negative bacteria is mainly recognized by cells of the innate immune response to modulate the inflammatory response (12), Gram-positive bacteria could exacerbate inflammation through other pathways, such as TLR2 (42). This study confirms that the presence of the A/G genotype of the SNP A-2570G of the *TLR4* gene is a risk factor in the presence of bacteria such as *C. acnes* (3.7), *S. anginosus* (3.7), and *S. gordonii* (2.5) for developing periodontitis.

Obesity is a low-grade chronic inflammatory disease, as is nasal polyposis (43), which has been associated with the A/G genotypes of the SNP A896G and the C/T of the SNP C1196T of the *TLR4* gene. However, bacterial infectious diseases such as urinary tract infections (UTIs) (21) or experimental exposure to LPS have not been related to these SNPs (12) or our results.

The linkage disequilibrium and haplotype frequency of the *TLR4* gene have been poorly studied. In diseases such as obesity, a high frequency of haplotypes such as AGAC, GGAC, and AAAC has been observed, which correspond to SNPs of the *TLR4* gene (A-2570G position 1, G-2081A position 2, A896G position 3, and C1196T position 4). In this study, the similarities in high frequency corresponded to the AAC haplotype of the *TLR4* gene (A896G position 1, A-2570G position 2, and C1196T position 3). Haplotypes such as AAT, AGT, and GAC were not found in studies on diseases such as obesity (40).

It has previously been found that presenting with the G allele of SNP A-2570G represented a risk factor for periodontitis. When analyzing the genotypic frequency, we observed that the AAAGCT haplogenotype was associated with the disease, but it did not represent a risk effect. However, the AAAGCC haplogenotype had a 2.3 times higher risk of developing periodontitis; both genotypes can occur in polymorphic heterozygous individuals for SNP A-2570G of the *TLR4* gene. The genotypic analysis shows us results that until now have not been evaluated or published.

Haplotypes with high LD tend to be inherited together; this allows for the possibility of using them as genetic markers to identify polymorphisms, as ion the case of the blocks between polymorphisms A896G-A-2570G and A-2570G-C1196T, which, according to the linkage disequilibrium coefficient in our study, have a high LD of >80. Therefore, there is a greater probability of finding the G allele of SNP A896G and the G allele of SNP A-2570G together and frequently in our population compared to the T allele of SNP C1196T.

Individual genetic variability and various environmental factors (including oral hygiene habits, diet, and tobacco use) could not be fully controlled in this study. These elements may have influenced the composition of the oral microbiota and the clinical manifestation of periodontal disease, thereby affecting the interpretation of the association with *TLR4* gene polymorphisms. Nevertheless, acknowledging these limitations is essential to properly contextualize the findings and to guide future research that explores these variables in greater depth.

## Conclusion

The agonistic or antagonistic biological effect of each bacterial species depends on the genotype present in each individual and the processes of destruction of dental support tissues. *C. acnes* represents a risk factor for developing periodontitis in individuals with the A/G genotype of SNP-2570, a genotype which is correlated with greater clinical attachment loss and periodontal pocket depth.

## Data Availability

Existing datasets are available in a publicly accessible repository: Publicly available datasets were analyzed in this study. This data can be found here: [http://www.ri.uagro.mx/handle/uagro/4927].
